# Genetic Diversity, Selection Signatures, and Genome-Wide Association Study Identify Candidate Genes Related to Litter Size in Hu Sheep

**DOI:** 10.3390/ijms25179397

**Published:** 2024-08-29

**Authors:** Jingjing Bao, Jinke Xiong, Jupeng Huang, Peifu Yang, Mingyu Shang, Li Zhang

**Affiliations:** 1 State Key Laboratory of Animal Biotech Breeding, Institute of Animal Science, Chinese Academy of Agricultural Sciences (CAAS), Beijing 100193, China; baojingjing720@163.com (J.B.); jinkexiong2021@163.com (J.X.); h18766816604@163.com (J.H.); caasyangpeifu@163.com (P.Y.); 82101211202@caas.cn (M.S.); 2 Key Laboratory of Animal Genetics, Breeding and Reproduction of Shaanxi Province, College of Animal Science and Technology, Northwest A&F University, Yangling 712100, China

**Keywords:** Hu sheep, genetic diversity, selection signatures, GWAS, litter size, candidate genes

## Abstract

Hu sheep is a renowned prolific local sheep breed in China, widely distributed across the country due to its excellent reproductive performance. Deciphering the molecular mechanisms underlying the high fecundity of Hu sheep is crucial for improving the litter size of ewes. In this study, we genotyped 830 female Hu sheep using the Illumina OvineSNP50 BeadChip and performed genetic diversity analysis, selection signature detection, and a genome-wide association study (GWAS) for litter size. Our results revealed that the Hu sheep population exhibits relatively high genetic diversity. A total of 4927 runs of homozygosity (ROH) segments were detected, with the majority (74.73%) being short in length. Different genomic inbreeding coefficients (*F*_ROH_, *F*_HOM_, *F*_GRM_, and *F*_UNI_) ranged from −0.0060 to 0.0126, showing low levels of inbreeding in this population. Additionally, we identified 91 candidate genomic regions through three complementary selection signature methods, including ROH, composite likelihood ratio (CLR), and integrated haplotype score (iHS), and annotated 189 protein-coding genes. Moreover, we observed two significant SNPs related to the litter size of Hu sheep using GWAS analysis based on a repeatability model. Integrating the selection signatures and the GWAS results, we identified 15 candidate genes associated with litter size, among which *BMPR1B* and *UNC5C* were particularly noteworthy. These findings provide valuable insights for improving the reproductive performance and breeding of high-fecundity lines of Hu sheep.

## 1. Introduction

Sheep are important economic animals, and their reproductive traits directly impact the production efficiency and economic benefits of the sheep industry [1]. Litter size is a crucial reproductive trait in sheep. Increasing litter size can rapidly expand the sheep population, thereby providing more high-quality mutton, wool, and milk products to meet market demand. Hu sheep, a renowned native Chinese sheep breed, stands out for its early sexual maturity, year-round estrus, high fecundity, rapid growth and development, good meat production performance, strong adaptability, rough feeding tolerance, and suitability for house feeding [2]. Due to its excellent characteristics and prominence as a leading agricultural breed in China, Hu sheep are widely distributed throughout the country. As one of the most prolific sheep breeds globally, Hu sheep ewes can produce lambs three times every two years, usually producing 2–3 lambs per litter, and occasionally up to 7 or 8 lambs [3,4]. Therefore, identifying the key genes underlying high prolificacy is of significant importance for optimizing breeding strategies and improving the reproductive performance of Hu sheep.

Runs of homozygosity (ROH) are continuous homozygous segments in the genome [5]. ROH formation is primarily associated with inbreeding, genetic drift, and selective pressures [6,7]. Long ROH segments are often related to inbreeding, while widely distributed short ROHs may indicate ancient common ancestors or historical events [8]. The length and number of ROH can be used to assess the degree of inbreeding and genetic diversity within a population. Evaluating genomic inbreeding levels based on ROH is generally considered the most accurate method [9,10]. Recently, ROH has been widely utilized as an important tool for identifying candidate genes and selection signatures linked to economic traits in livestock [11,12,13,14,15]. Through ROH analysis, we can better understand the genetic diversity, levels of inbreeding, and candidate genes associated with important economic traits in the Hu sheep population.

The genetic markers or characteristics left on the genome due to artificial or natural selection are known as selection signatures [16]. Detecting these selection signatures can provide valuable information for exploring the genetic background of animals, the formation of economic traits, adaptability, and domestication [17]. There are various methods for detecting selection signatures within populations, categorized primarily into the following three types: (1) methods based on reduction in genetic variability, such as ROH [18] and pooled heterozygosity (Hp) [19]; (2) summary statistics based on site frequency spectrum (SFS), including Tajima’s D [20] and composite likelihood ratio (CLR) [21]; and (3) methods based on haplotype information, such as extended haplotype homozygosity (EHH) [22] and integrated haplotype score (iHS) [23]. Comprehensively utilizing all genomic feature information can more accurately uncover selection signatures across the genome. Nayak et al. [24] used four intra-population statistics (Tajima’s D, CLR, iHS, and ROH) to uncover climate resilience in Indian cattle breeds. Zhao et al. [25] employed three complementary approaches (CLR, iHS, and ROH) to detect the selection signatures related to breed-specific traits in Hu sheep.

The genome-wide association study (GWAS) is an important method for identifying major genes linked to traits [26]. Fertility is a critical economic trait in sheep breeding and production. A series of genetic loci significantly associated with the reproductive traits of sheep have been identified using GWAS [27,28,29], and the key genes such as *BMPR1B* [30,31,32], *GDF9* [33,34], and *BMP15* [35] mainly influence litter size by affecting the ovulation rate. In this study, based on the Illumina OvineSNP50 BeadChip data and phenotypic information of litter size, we investigated the genetic diversity, inbreeding levels, and combined selection signatures and GWAS analyses to identify candidate genes linked to the litter size of Hu sheep. This research provides important support for elucidating the genetic mechanisms underlying the prolificacy and breeding of high-fertility lines of Hu sheep.

## 2. Results

### 2.1. Principal Component Analysis and Genetic Diversity Estimation

**Figure 1:**
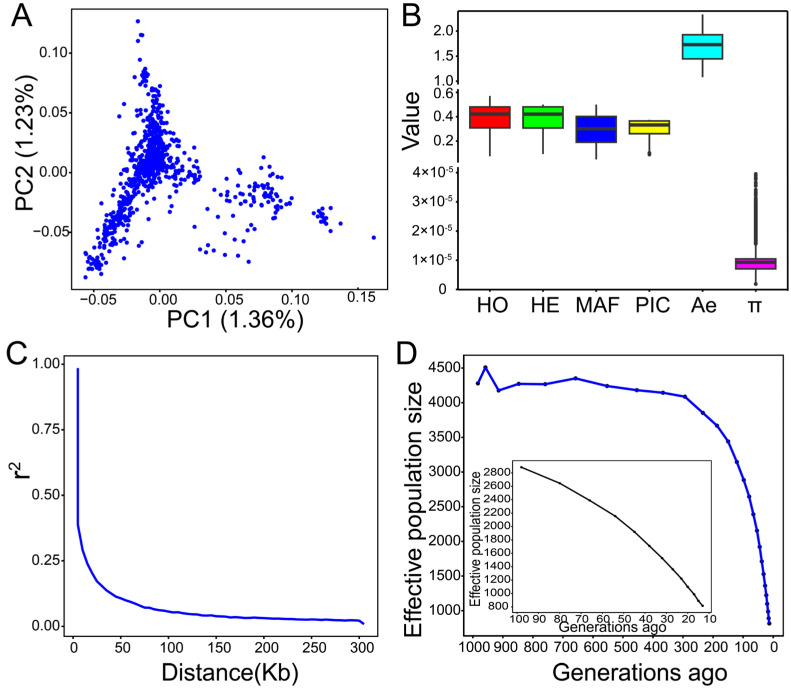
PCA and genetic diversity analysis of female Hu sheep population. (**A**) PCA of the Hu sheep population; (**B**) Box plot of genetic diversity indices of Hu sheep. HO, observed heterozygosity; HE, expected heterozygosity; MAF, minor allele frequency; PIC, polymorphism information content; Ae, effective number of alleles; π, nucleotide diversity; (**C**) LD decay plot of Hu sheep; (**D**) The estimated effective population size of Hu sheep.

As shown in Figure 1A, the results of principal component analysis (PCA) indicated no significant stratification in the female Hu sheep population. The first two principal components (PCs) explained 1.36% and 1.23% of the variation, respectively.

To assess the genetic diversity of the Hu sheep population, seven estimated indicators were demonstrated in Figure 1B and Table S1. Observed heterozygosity (HO) ranged from 0.07 to 0.57, while expected heterozygosity (HE) spanned from 0.09 to 0.5, with the average HO (0.39) slightly exceeding HE (0.38). The average minor allele frequency (MAF) was 0.29, varying between 0.05 and 0.50. The proportion of polymorphic markers (PN) was 0.91, and the mean polymorphism information content (PIC) was 0.30, with 77.35% of SNPs falling between 0.25 and 0.50, indicating a moderate level of polymorphism in the ewe population. The average effective number of alleles (Ae) was 1.68, and the nucleotide diversity (π) was 9.93 × 10^−6^. The LD coefficient r^2^ of Hu sheep gradually decreased as the physical distance between SNPs increased (Figure 1C). When the LD decay distance was 5 kb, the average r^2^ reached its highest value (0.99). Subsequently, the r^2^ rapidly decreased, falling below 0.1 at an average LD decay distance of 50 kb. Figure 1D illustrates the overall trend of the estimated effective population size (Ne) of Hu sheep decreasing over time. Ne was estimated as 816 as of 13 generations ago and 4279 as of 983 generations ago.

### 2.2. Distribution of Runs of Homozygosity

**Figure 2:**
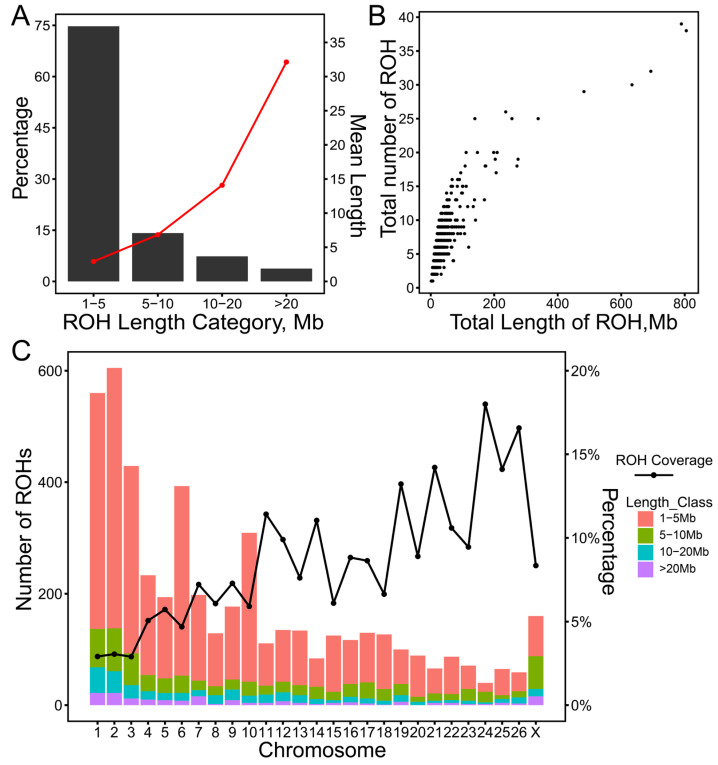
Distribution of ROHs in the Hu sheep population. (**A**) The frequency (bars) and average length (red line) of ROHs in different length categories; (**B**) The total length of ROHs and the total number of ROHs for per sheep; (**C**) The number of ROHs of different ROH length categories on each chromosome (bars) and the percentage of each chromosome covered by ROHs in the Hu sheep population (black line).

A total of 4927 ROHs were identified from 816 ewes, while no ROHs were detected in another 14 sheep. The length of the ROHs ranged from 1.55 Mb to 87.64 Mb, with an average ROH length of 5.39 Mb. The majority of ROHs were between 1 and 5 Mb in length, representing 74.73% of the total (Figure 2A). Additionally, the frequencies of the ROHs with lengths of between 5 and 10 Mb, 10 and 20 Mb, and >20 Mb were 14.17%, 7.35%, and 3.76%, respectively. The average length of the ROHs per animal within each ROH category spanned from 2.91 Mb to 32.13 Mb. Descriptive statistics for the four different length categories of ROHs are presented in Table S2. In general, the total length of ROHs per animal increased with the number of ROHs. As depicted in Figure 2B, the longest total length of ROHs was 804.96 Mb, consisting of 38 ROHs, while the shortest was 1.87 Mb, comprising only 1 ROH. Figure 2C shows the number of ROHs and ROH coverage on each chromosome in the Hu sheep population. The largest number of ROHs was observed on chromosome 2 (605 ROHs), accounting for approximately 12.28% of the total number of identified ROHs. In contrast, chromosome 24 had the fewest number of ROHs (40 ROHs), representing about 0.81% of the total ROHs. Chromosome 3 exhibited the lowest ROH coverage (2.90%), while chromosome 24 had the highest ROH coverage (18.00%) (Figure 2C).

### 2.3. Estimation of Inbreeding Coefficients

The inbreeding coefficient based on the total length of the ROHs (*F*_ROH total_) ranged from 0.0007 to 0.3119, with an average value of 0.0126. Notably, among the estimated inbreeding coefficients based on the different ROH lengths, the average *F*_ROH>20Mb_ (0.0295) was significantly higher than *F*_ROH 1–5Mb_ (0.0051), *F*_ROH 5–10Mb_ (0.0049), and *F*_ROH 10–20Mb_ (0.0091). In this study, the average values of *F*_HOM_ (−0.0060), *F*_GRM_ (−0.0055), and *F*_UNI_ (−0.0055) were all negative. Summary statistics for all estimated inbreeding coefficients are presented in Table S3.

**Figure 3:**
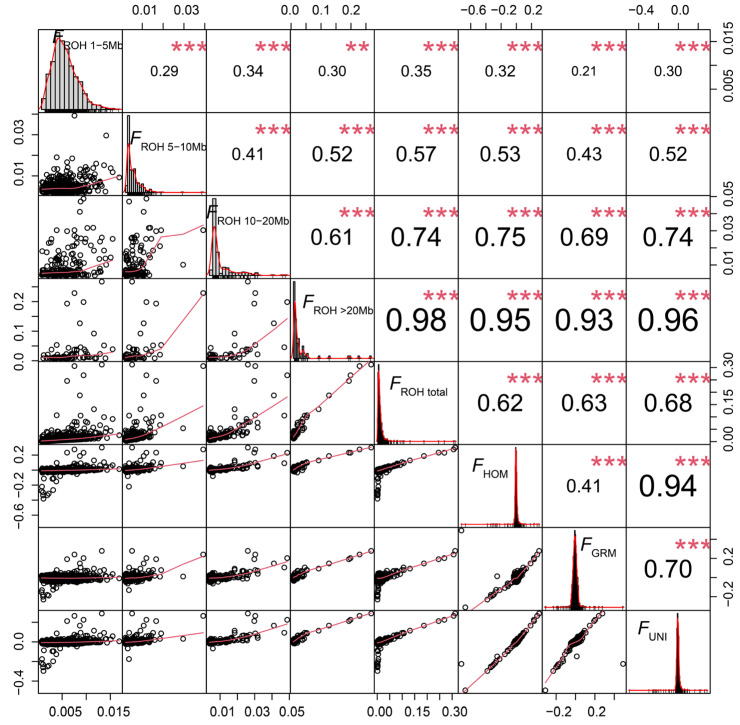
Scatterplots (bottom left) and Pearson’s correlations (top right) for eight different inbreeding coefficients in Hu sheep. ** denotes *p*-value < 0.01, *** denotes *p*-value < 0.001.

Pearson’s correlations among eight kinds of genomic inbreeding coefficients are displayed in Figure 3. The inbreeding coefficients of Hu sheep obtained by different methods all showed extremely significant positive correlations (*p* < 0.01). Among them, the correlation between *F*_ROH total_ and *F*_ROH>20Mb_ was the strongest (r = 0.98), while the weakest correlation was between *F*_ROH 1–5Mb_ and *F*_GRM_ (r = 0.21). The correlation between *F*_HOM_ and *F*_UNI_ was also high (r = 0.94).

### 2.4. Detection of Selection Signatures

**Figure 4:**
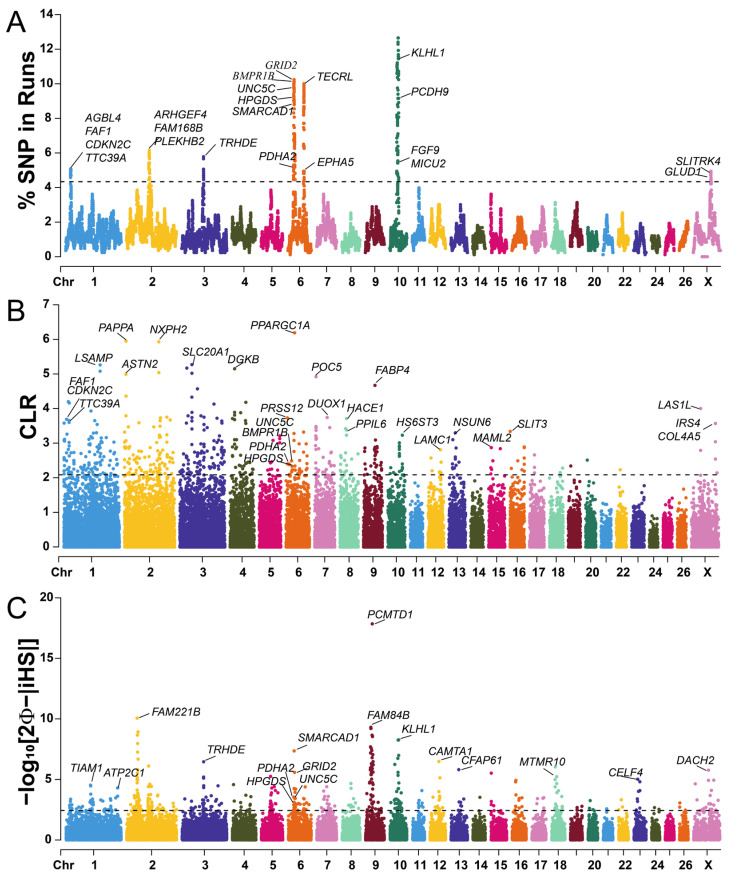
Manhattan plots of selection signatures detected by ROH (**A**), CLR (**B**), and iHS (**C**) methods in Hu sheep. The black dashed lines indicate the top 1% threshold.

Eight high-frequency ROH islands was identified, distributed on chromosomes 1, 2, 3, 6, 10, and X, containing 468 SNPs (Figure 4A). Interestingly, the majority of ROH islands are located on chromosomes 6 and 10, which may be most relevant to the breed-specific traits in Hu sheep. Figure 4B displays the distribution of CLR values across the Hu sheep genome. Based on the top 1% of CLR values, a total of 278 candidate regions were selected. Additionally, we detected 237 selection signatures on all chromosomes according to the iHS test, and the highest *p*_iHS_ value was observed on chromosome 9 (Figure 4C).

**Figure 5:**
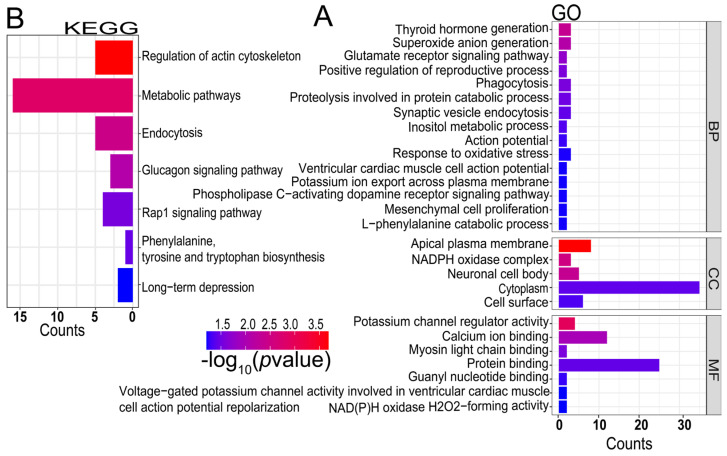
Functional enrichment analysis of candidate genes simultaneously identified by at least two selection signature methods in Hu sheep. (**A**) GO. (**B**) KEGG.

A total of 91 candidate genomic regions were simultaneously detected using at least two methods, and 189 protein-coding genes were annotated (Table S4). Among them, six common overlapping regions were identified by the ROH, CLR, and iHS tests and were distributed on chromosomes 1, 3, and 6. These candidate genes significantly enriched in 27 Gene Ontology (GO) terms, such as thyroid hormone generation (GO: 0006590), positive regulation of reproductive process (GO:2000243), mesenchymal cell proliferation (GO:0010463), phospholipase C-activating dopamine receptor signaling pathway (GO:0060158), and guanyl nucleotide binding (GO:0019001) are linked to reproductive traits (Figure 5A, Table S5). Seven Kyoto Encyclopedia of Genes and Genomes (KEGG) pathways were significantly enriched, including the regulation of the actin cytoskeleton (oas04810), metabolic pathways (oas01100), endocytosis (oas04144), glucagon signaling pathway (oas04922), rap1 signaling pathway (oas04015), phenylalanine, tyrosine and tryptophan biosynthesis (oas00400), and long-term depression (oas04730) (Figure 5B, Table S5). We found these candidate genes associated with litter size (*FAF1*, *CDKN2C*, *PDHA2*, *BMPR1B*, *UNC5C*, *HPGDS*, *SMARCAD1*, *DUOX1*, *CTSK*, *DUOX2*, *FGF7*, *FGF9*, *GNA14*, *GNAQ*).

### 2.5. GWAS for Litter Size of Hu Sheep

**Figure 6:**
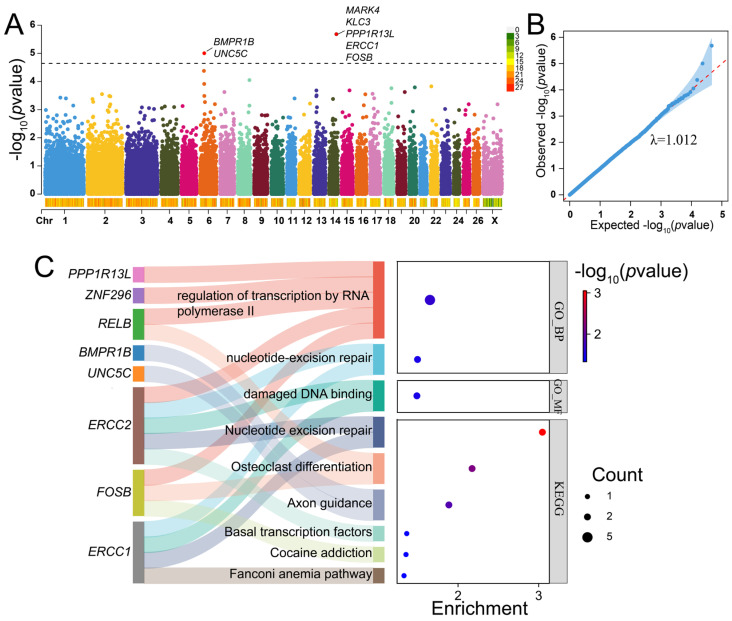
The results of GWAS for litter size of Hu sheep. (**A**) Manhattan plot of the GWAS for litter size in Hu sheep. The black dashed line indicates the thresholds for litter size in Hu sheep (−log10(*p*-value) = 4.64). (**B**) QQ plot of GWAS for litter size in Hu sheep. (**C**) Sankey and dot plots showing the significantly enriched GO terms and KEGG pathways connected to candidate genes identified by GWAS for litter size in Hu sheep.

A total of 2068 litter records from 830 ewes across parities 1 to 8 were collected, with an average litter size of 2.36 (Table S6). The results of the GWAS for the litter size of Hu sheep are shown in Figure 6 and Table S7. Two significant SNPs were identified, one is *FecB* located on chromosome 6, annotated to the *BMPR1B* gene, a major gene associated with the litter size of sheep, and the other on chromosome 14 was annotated to *MARK4* (Figure 6A). The QQ plot for litter size showed a very slight genomic inflation factor with λ_gc_ = 1.012 (Figure 6B). We annotated 27 candidate protein-coding genes within 250 kb upstream and downstream of the significant SNPs (Table S7). Candidate genes were significantly enriched in three GO terms, including the regulation of transcription by RNA polymerase II (GO:0006357), nucleotide-excision repair (GO:0006289), and damaged DNA binding (GO:0003684) (Figure 6C, Table S8). Furthermore, we obtained six significant KEGG pathways, one of them was axon guidance (oas04360), containing two genes (*UNC5C*, *BMPR1B*) and closely related to litter size (Figure 6C, Table S8). Interestingly, *BMPR1B* and *UNC5C* were also identified in the above selection signature methods.

## 3. Discussion

### 3.1. PCA and Genetic Diversity Analysis

The analysis of population structure and genetic diversity provides important insights for breed conservation and the genetic improvement of Hu sheep. In this study, the first two principal components explained 2.59% of the total variance in the female Hu sheep population. The results indicate that there is no significant population stratification, and individuals within the population may have very similar genetic backgrounds.

We conducted a comprehensive evaluation of genetic diversity in Hu sheep by calculating seven parameters (HO, HE, MAF, PN, PIC, Ae, and π). Heterozygosity is one of the most important parameters for estimating population genetic variability. Our studies found that the HO value (0.39) is slightly higher than the HE (0.38), which shows a higher level of genetic diversity within the Hu sheep population and suggests that the population may be independent of inbreeding. Additionally, MAF is widely utilized in genetic diversity studies to distinguish common and rare variants within populations. Heaton et al. [36] reported that SNPs with a MAF value greater than or equal to 0.3 are considered highly informative. In the Hu sheep population, the average MAF was 0.29, with about 50.48% of SNPs being highly variable (MAF ≥ 0.3). The PN value of 0.91 indicates that the sheep population has an abundance of polymorphic SNP sites. PIC is a commonly used and effective indicator for measuring genetic polymorphism of molecular markers. This ewe population presented moderate genetic polymorphism (PIC = 0.30). Nucleotide diversity represents the genetic diversity of the population to some extent. The sheep population may have undergone selection pressure that made certain high-frequency variants more common, resulting in lower overall nucleotide diversity (π = 9.93 × 10^−6^) [37]. The effective number of alleles also provides additional information regarding genetic diversity.

The pattern and rate of linkage disequilibrium (LD) decay reveal the population history. Specifically, when a population undergoes expansion, the rate of LD decay may accelerate due to an increase in the opportunities for genetic recombination, thereby enhancing the genetic diversity of the population. Conversely, if a population experiences a bottleneck effect, the rate of LD decay may slow down [38]. Our observed pattern of LD decay is consistent with findings from previous studies on sheep [39,40]. The degree of LD between SNPs can be used to infer the ancestral effective population size (Ne), which is a key parameter for assessing population genetic diversity. Generally, smaller values of Ne indicate reduced genetic variation within populations. The Ne reported in our study is notably larger than in other studies and is closer to the values analyzed in most sheep breeds in the SheepHapMap project (Spanish sheep with Ne around 600, Finnish sheep with Ne around 795) [41]. This difference may be attributed to the SheepHapMap project’s utilization of fewer and unrelated animals, while other studies have mostly involved some degree of related animals. Overall, the sheep population has relatively high genetic diversity, which provides a favorable basis for the protection and utilization of Hu sheep.

### 3.2. Characteristics of Runs of Homozygosity

The formation of an ROH can occur in multiple ways, including recent inbreeding, genetic drift, selection pressures, and random events during genomic recombination [8,18]. By detecting the length, number, and distribution of the ROHs, we can assess the level of inbreeding, analyze genetic diversity within populations, and identify the impacts of genetic bottlenecks and drift events. In this study, the identified ROHs were predominantly short segments (1–5 Mb). This indicates a low level of breeding among these ewes which may not have experienced significant recent inbreeding events. Additionally, these factors such as larger effective population size, lower genetic drift, higher genomic recombination rates, and the influence of selection pressure may contribute to the high frequency of short ROH segments.

Previous studies have shown that ROHs tend to be enriched on specific chromosomes, and the number of ROHs is related to the physical length of the chromosome [42]. Our study found that the top five highest numbers of ROHs were distributed on chromosomes 1, 2, 3, 6, and 10, which is consistent with the findings of Li et al. in Hu sheep [4]. Notably, chromosomes 1, 2, and 3 are the largest metacentric sheep chromosomes by far. We observed that SNPs with the highest frequency of occurrence are primarily located on chromosomes 6 and 10, and the previous reports have indicated that these chromosomes are rich in candidate genes associated with the reproduction of Hu sheep [4,25].

### 3.3. Genomic Inbreeding Coefficients Estimation

Traditional inbreeding coefficients are calculated based on pedigree information. Pedigrees may contain errors or incomplete records, leading to the inaccurate estimation of inbreeding levels. In contrast, genomic inbreeding coefficients calculated using SNP molecular markers can resolve the above problems. Theoretically, the inbreeding coefficient should range between 0 and 1 [43]. In our study, *F*_HOM_, *F*_GRM_, and *F*_UNI_ were negative values. This may be due to the presence of a heterozygote advantage for certain genotypes in the Hu sheep population, which increases the probability of heterozygotes, leading to a lower observed proportion of homozygous loci than expected. However, *F*_ROH_ is based on continuous homozygous fragments in the genome, and its estimated values are always positive. Therefore, *F*_ROH_ is a more effective and accurate method for quantifying inbreeding levels [8,44]. Additionally, the short segments of ROH were more prevalent in the Hu sheep population, but the inbreeding coefficient based on long segments of ROH was greater than those based on the short segments of ROH, and it had the strongest correlation with the inbreeding coefficient calculated based on the total ROH length. This may be because the selection pressure which affects certain individuals in the Hu sheep population, resulting in longer homozygous regions in some genomic regions. There are highly significant positive correlations between the different genomic inbreeding coefficients, indicating their overall consistency and the general prevalence of inbreeding patterns. In summary, using multiple inbreeding metrics provides a more comprehensive understanding of the inbreeding levels in the Hu sheep population.

### 3.4. Candidate Genes Associated with Litter Size Trait

Hu sheep is a renowned prolific breed in China, and identifying candidate genes related to litter size can provide valuable information for breeding and enhancing production performance. In this study, through the combined analysis of selection signatures and GWAS, we identified two key genes (*BMPR1B*, *UNC5C*) associated with litter size in Hu sheep. *BMPR1B*, an essential member of the bone morphogenetic protein (*BMP*) receptor family, is a major gene affecting the litter size in sheep [45]. It plays an important role in follicular development and mainly increases the litter size by increasing the number of ovulations in the ovaries of ewes [46]. Many studies have reported that the *BMPR1B* gene is associated with the litter size of Hu sheep [4,25,47,48,49,50]. Our GWAS analysis identified a single significant SNP on chromosome 6, the *FecB* mutation, which is located within the exon of *BMPR1B*. This mutation has an additive effect on the ovulation rate in sheep, increasing the number of ovulations by an additional 1.5 to 3.0 per gene copy [51]. As a result, the *FecB* mutation has become one of the marker genes for high fecundity in sheep and is widely utilized in the breeding of prolific sheep. *UNC5C* is a protein-coding gene, and its product belongs to the UNC-5 family of netrin receptors. Variants within *UNC5C* have been found to have a correlation with the conception rate in cattle [52], and whole-genome resequencing revealed that the *UNC5C* gene was linked to litter size in Hu sheep [25,48,50,53].

We simultaneously found five additional candidate genes linked to reproductive traits by employing three complementary statistics (ROH, CLR, and iHS). *FAF1*, a member of the *Fas* family, may be closely related to the development of testicular and ovarian tissues in domestic yarks [54]. *CDKN2C* is differentially expressed in the testis of male infertility in cattleyak [55] and is involved in testis development and spermatogenesis in mice [56]. *PDHA2*, a testis-specific gene in humans, encodes the pyruvate dehydrogenase complex E1α subunit involved in cellular energy metabolism and regulates germ cell development, spermatid differentiation, and spermatogenesis [57]. A GWAS analysis of semen traits identified the candidate gene *HPGDS*, which negatively regulates male germ cell proliferation [58]. *SMARCAD1*, differentially expressed between fertile and infertile spermatozoa, has been suggested to have a significant role in spermatogenesis and male fertility [59].

In addition, some genes regulate reproductivity related signaling pathways to influence litter size. *DUOX1*, *DUOX2*, and *CTSK* are enriched in the thyroid hormone generation pathway, impacting gonadal function, sex hormone metabolism, and the hypothalamic–pituitary–gonadal axis activity. *DUOX1* and *DUOX2* have been reported to be relevant to spermatogenesis and male fertility [60]. *CTSK* is closely related to sheep ovulation [61]. *FGF9* and *UNC5C* are involved in the positive regulation of reproductive processes. *FGF9* may be related to high fertility in Hu sheep by affecting spermatogenesis, and ovarian and embryo development [4]. The biological process of mesenchymal cell proliferation (*FGF7*, *FGF9*) positively affects litter size by influencing ovarian function, sperm formation, and embryo development. *FGF7* participate in the follicles and ovary development in Hu sheep [62]. *GNA14* and *GNAQ* are enriched in the guanyl nucleotide-binding and phospholipase C-activating dopamine receptor signaling pathway. *GNA14* and *GNAQ* belong to the G protein α-subunit family; *GNAQ* gene may affect GnRH secretion to regulate sheep seasonal estrus [63].

Notably, the most significant SNP in the GWAS for litter size in Hu sheep is located within the intron region of the *MARK4* gene. This gene encodes a member of the microtubule affinity-regulating kinase (*MARK*) family and may be involved in cell cycle control. *MARK4*, which exhibits the highest expression in the testis, is a component of ectoplasmic specialization. It regulates the sertoli cell blood–testis barrier dynamics through microtubule and actin cytoskeletons and plays an important role in spermatogenesis in the rat testis [64,65]. Additionally, *MARK4* expression is downregulated in women with ovulatory polycystic ovary syndrome, which inhibits follicular development and the ovulation process [66]. These findings suggest that *MARK4* may be a key genetic marker influencing the litter size in Hu sheep through the regulation of reproductive processes.

## 4. Materials and Methods

### 4.1. Sampling and DNA Extraction

A total of 830 female Hu sheep, born between 2017 and 2021, were randomly selected from the core flock at Gansu Zhongsheng Huamei Sheep Industry Development Co., Ltd. (Qingyang, China), a national sheep core breeding farm that raises approximately 6000 breeding ewes. Whole blood samples were collected from the jugular vein of each ewe using 5 mL blood collection tubes containing ethylenediaminetetraacetic acid (EDTA) and stored at −20 °C. Genomic DNA was extracted from blood samples using the magnetic bead method according to the manufacturer’s instructions for the Maghead Blood DNA Kit (Kangwei Century Biotechnology Co., Ltd., Beijing, China).

### 4.2. Genotyping and Quality Control

Qualified genomic DNA was genotyped with the Illumina OvineSNP50 BeadChip (Illumina, San Diego, CA, USA). All SNP positions were mapped on the sheep reference genome Oar_v4.0. SNPs meeting the following criteria were excluded using PLINK v1.90 software [67]: (1) SNP call rate < 0.95; (2) minor allele frequency (MAF) < 0.05; (3) Hardy–Weinberg equilibrium (HWE) < 1 × 10^−6^. SNPs located on autosomes and the X chromosome were included. After quality control, 46,371 SNPs and 830 individuals were retained. Finally, Beagle v5.2 software [68] was employed to impute missing genotypes.

### 4.3. Principal Component Analysis and Genetic Diversity

To assess the genetic relationships among individuals in the Hu sheep population, we performed principal component analysis (PCA) using GCTA v1.94.1 [69] and visualized the results with the R package “ggplot2” v3.5.0. Genetic diversity indices, including observed heterozygosity (HO), expected heterozygosity (HE), minor allele frequency (MAF), polymorphism information content (PIC), effective number of alleles (Ae), and proportion of polymorphic markers (PN), were calculated using PLINK v1.90 [67]. Additionally, VCFtools v0.1.16 [70] was utilized to evaluate nucleotide diversity (π) with a non-overlapping 50 kb window. Linkage disequilibrium (LD) decay was determined using PopLDdecay v3.42 [71]. The effective population size (Ne) was estimated using SNeP v1.1 [72] with default parameters.

### 4.4. Runs of Homozygosity Detection

ROHs were identified using the R package “detectRUNS” v0.9.6 with the following parameters: (1) allowance for at most one heterozygous SNP and one missing genotype; (2) the maximum gap between consecutive SNPs and the minimum ROH length was both set to 1 Mb; (3) the minimum SNP density was 1 SNP every 500 kb. The minimum number of homozygous/heterozygous SNP in the window was calculated using the following formula [73]:

$$l=\frac{ln\frac{\alpha }{n_{s}*n_{i}}}{ln(1-het)},$$

where α is the percentage of false positive ROHs and is set to 0.05, *n_s_* is the number of SNPs each sheep, *n_i_* is the number of animals, and *het* is the average SNP heterozygosity across all SNPs. The ROH lengths were classified into the following four categories: 1–5 Mb, 5–10 Mb, 10–20 Mb, and >20 Mb.

### 4.5. Inbreeding Coefficients Estimation

Eight different methods were used to estimate the genomic inbreeding coefficient among individuals. The first five methods were based on the proportion of the ROH length to the genome length using the “detectRUNS” package, including *F*_ROH total_, *F*_ROH 1-5 Mb_, *F*_ROH 5–10Mb_, *F*_ROH 10–20Mb_, and *F*_ROH>20Mb_. The sixth method was based on the excess of homozygosity (*F*_HOM_) using PLINK v1.90 [67] with the option “-het”. The last two methods were, respectively, based on the genomic relationship matrix (*F*_GRM_) and correlation between uniting gametes (*F*_UNI_) using GCTA v1.94.1 [69]. Pearson’s correlation coefficients were calculated and visualized using the “chart.Correlation” function from the R package “PerformanceAnalytics” v2.0.4 to compare the genomic inbreeding coefficients estimated by the above methods.

### 4.6. Selection Signatures Identification

To more comprehensively and accurately identify selection signatures in the Hu sheep population, we employed three complementary methods—ROH, CLR, and iHS. High-frequency ROH regions were identified based on the proportion of times each SNP appeared in the ROHs in the Hu sheep population. SNPs with the top 1% occurrence frequency were selected, and adjacent SNPs were merged to form ROH islands. For the CLR test, SweeD v4.0.0 [74] software was used to calculate CLR values in 50 kb windows. Windowsat the top 1% of ranked likelihood values were regarded as selected regions. iHS values for each SNP were calculated using the R packages “rehh” v3.2.2 [75] and standardized according to the following formula [76]:

$$p_{iHS}=-{log}_{10}(1-2|\Phi \left(iHS\right)-0.5|)$$

where Φ(x) represents the Gaussian cumulative distribution function. Then, a windowed iHS test was performed with a window size of 500 kb and a 100 kb overlap, and a threshold based on the top 1% of $p_{iHS}$ values. Genomic regions detected by at least two methods were considered candidate selection signatures.

### 4.7. Genome-Wide Association Study

We conducted GWAS on the litter size of ewes using GMATs v1.01 [77] software based on an animal repeatability model. The model was as follows:

y=Wα+Xβ+Zμ+e

where y is the litter size, α includes vectors of fixed effects (parity, years, and seasons), β is the vector of random effects, μ is the vector of permanent environmental effects, e is the vector of residual effects, and W, X, and Z are the correspondent incidence matrices of α, β, and μ, respectively. In this study, we used a false discovery rate (FDR = 0.005) to determine the threshold *p* values of GWAS [40]. Manhattan and QQ plots were visualized in the R package “CMplot” v4.5.1.

### 4.8. Gene Annotation and Functional Enrichment Analysis

Gene annotation was performed on the candidate selection signatures and regions of 250 kb upstream and downstream of the significant SNPs from GWAS using BEDTools v2.25.0 [78] software. To further understand the biological functions of the candidate genes, we performed Gene Ontology (GO) and Kyoto Encyclopedia of Genes and Genomes (KEGG) pathway enrichment analysis using the DAVID tool (https://david.ncifcrf.gov/, accessed on 22 July 2024) and the KOBAS tool (http://bioinfo.org/kobas/, accessed on 22 July 2024), respectively.

## 5. Conclusions

This study offers a comprehensive genomic analysis of high fecundity in Hu sheep based on the Illumina OvineSNP50 BeadChip data and phenotypic data of litter size. Our results demonstrate a high level of genetic diversity in the Hu sheep population, indicating substantial potential for improving litter size. Through the combined analysis of selection signatures and GWAS, we identified several candidate genes associated with litter size (*FAF1*, *CDKN2C*, *PDHA2*, *UNC5C*, *BMPR1B*, *HPGDS*, *SMARCAD1*, *DUOX1*, *CTSK*, *DUOX2*, *FGF7*, *FGF9*, *GNA14*, *GNAQ*, and *MARK4*). Among them, *BMPR1B* and *UNC5C* were the most promising candidate genes for the litter size in Hu sheep. Further functional validation of the candidate genes is needed to clarify their specific roles in the litter size. These findings provide valuable genetic markers and candidate genes for breeding programs aimed at improving the reproductive performance of Hu sheep.

## Data Availability

The data used in this study are available from the corresponding author upon reasonable request.

## Supplementary Materials

The following supporting information can be downloaded at: https://www.mdpi.com/article/10.3390/ijms25179397/s1.
