# Supplementary material for: Genetic Diversity, Selection Signatures, and Genome-Wide Association Study Identify Candidate Genes Related to Litter Size in Hu Sheep
Source: Int J Mol Sci. 2024 Aug 29;25(17):9397. doi: 10.3390/ijms25179397 (PMC11395453; doi:10.3390/ijms25179397)
Supplement: Supplementary file 1 [file ijms-25-09397-s001.zip › ijms-3169158-supplementary.pdf]

Table S1. The seven indicators of genetic diversity of female Hu sheep population.

Parameters	Mean
Heterozygosity Observed (HO)	0.39
Expected Heterozygosity (HE)	0.38
Minor Allele Frequency (MAF)	0.29
Proportion of Polymorphic Markers (PN)	0.91
Polymorphism Information Content (PIC)	0.30
Effective Numbers of Alleles (Ae)	1.68
nucleotide diversity (π)	9.93×10^{-6}

Table S2. Descriptive statistics of ROHs in four different length categories.

ROH Length (Mb)	ROH Number	Percentage (%)	Mean Length (Mb)
1-5	3682	74.73	2.91
5-10	698	14.17	6.83
10-20	362	7.35	14.09
>20	185	3.76	32.13
Total	4927	100	5.39

Table S3. Descriptive statistics for different inbreeding coefficients for the Hu sheep.

Inbreeding Coefficient	Mean	SD	Mix	Max
$F_{ROH\ 1\text{-}5Mb}$	0.0051	0.0027	0.0007	0.0169
$F_{ROH\ 5\text{-}10Mb}$	0.0049	0.0038	0.0019	0.0393
$F_{ROH\ 10\text{-}20Mb}$	0.0091	0.0079	0.0039	0.0488
$F_{ROH\ >20Mb}$	0.0295	0.0492	0.0078	0.2675
$F_{ROH\ total}$	0.0126	0.0238	0.0007	0.3119
F_{HOM}	-0.0060	0.0531	-0.723	0.2999
F_{GRM}	-0.0055	0.0379	-0.3114	0.4911
F_{UNI}	-0.0055	0.0387	-0.4944	0.2897

* $F_{ROH\ 1\text{-}5Mb}$, inbreeding coefficient estimated from ROH in length of 1-5 Mb; $F_{ROH\ 5\text{-}10Mb}$, inbreeding coefficient estimated from ROH in length of 5-10 Mb; $F_{ROH\ 10\text{-}20Mb}$, inbreeding coefficient estimated from ROH in length of 10-20 Mb; $F_{ROH\ >20Mb}$, inbreeding coefficient estimated from ROH longer than 20 Mb; $F_{ROH\ total}$, inbreeding coefficient estimated from the total ROH length; F_{HOM}, excess homozygosity estimator; F_{GRM}, inbreeding coefficient based on the diagonal elements of the genomic relationship matrix; F_{UNI}, inbreeding coefficient based on the correlation between uniting gametes; SD, standard deviation; Min, minimum; Max, maximum.

Table S4. Candidate genes annotated in overlapping regions simultaneously identified by at least two selection signature methods.

Chr	Start	End	Methods	Candidate Genes
1	21800000	22590130	CLR, iHS	*TRABD2B, LOC101114665, LOC101114922, SLC5A9, SPATA6*
1	23408141	24100000	ROH, iHS	*AGBL4, LOC105605154*
1	24796054	24999999	ROH, CLR	*FAF1*
1	25000000	25900000	ROH, CLR, iHS	*FAF1, CDKN2C, C1H1orf185, RNF11, TTC39A, EPS15, LOC105609515, OSBPL9*
1	25900001	26355803	ROH, CLR	*OSBPL9, NRDC, RAB3B, TXNDC12, KTI12, BTF3L4, LOC105609508,*

				LOC106990795, ZFYVE9
1	99500000	99843378	CLR, iHS	*ARNT, CTSS, CTSK, SETDB1, CERS2, FAM63A, ANXA9, PRUNE, BNIPL, C1H1orf56, CDC42SE1, GABPB2, SEMA6C, TNFAIP8L2, LYSMD1*
1	118900000	119633456	CLR, iHS	*RCAN1, KCNE1, SMIM11A, C1H21orf140, KCNE2, MRPS6, SLC5A3*
1	126853255	126897605	CLR, iHS	—
1	129576794	130000000	CLR, iHS	—
1	130200000	130629505	CLR, iHS	—
1	134800000	135282730	CLR, iHS	*LOC101120465, LOC105605728*
1	135365856	136239679	CLR, iHS	—
1	173130285	173356308	CLR, iHS	—
1	183100000	183577917	CLR, iHS	*GPR156, LRRC58, FSTL1, NDUFB4, HGD, RABL3, GTF2E1*
1	190000000	190882619	CLR, iHS	*BDH1, APOD, PPP1R2, ACAP2, XXYLT1*
1	194600000	194724026	CLR, iHS	—
1	210500000	211170136	CLR, iHS	*NAALADL2, NLGN1*
1	211256622	211400000	CLR, iHS	*NLGN1*
2	20539615	20871772	CLR, iHS	—
2	55365355	55901387	CLR, iHS	—
2	58317108	58400000	CLR, iHS	—
2	58500000	59313028	CLR, iHS	*GNAQ, GNA14, VPS13A*
2	62891541	63000000	CLR, iHS	—
2	63800000	64293321	CLR, iHS	*GDA, C2H9orf85, ABHD17B*
2	72200000	72491926	CLR, iHS	*SLC1A1, SPATA6L, PLPP6, CDC37L1*
2	110524110	110805843	CLR, iHS	*SH3RF1*
2	110991169	111400000	ROH, iHS	*LOC105611786, LOC101115261, ANXA10*
2	114400000	115657220	ROH, iHS	—
2	127164182	128000000	CLR, iHS	*ITGA4, UBE2E3*
2	154914678	155306560	CLR, iHS	—
2	162000000	162610701	CLR, iHS	—
2	178600000	179097069	CLR, iHS	*DPP10*
2	233993980	234000000	CLR, iHS	—
3	47701446	48400000	CLR, iHS	*LRRTM4*
3	48800000	48984677	CLR, iHS	
3	87400000	88143309	CLR, iHS	*LOC101122183, LOC105614651*
3	102600000	102687583	CLR, iHS	*CNGA3, VWA3B*
3	104211651	104500000	CLR, iHS	*PROM2, LOC106990992, ZNF2, ZNF514, MRPS5, LOC101117493, LOC101115527, MALL, NPHP1*
3	105500000	105982434	CLR, iHS	*ANAPC1, LOC105611974, MERTK, TMEM87B*
3	108207996	109005756	ROH, iHS	*TRHDE*
3	109005757	109100000	ROH, CLR, iHS	—
3	109100001	109119833	ROH, CLR	—
3	118400000	120400000	CLR, iHS	*TMTC2, LOC101104054, LOC105614909, LOC105614910, LOC105614711*

3	146179465	146450950	CLR, iHS	*MUC19, LRRK2*
3	171500000	171815525	CLR, iHS	*PAH, LOC101110666*
3	196736583	196983701	CLR, iHS	—
4	68201227	68477435	CLR, iHS	*JAZF1, TAX1BP1, HIBADH*
4	90160471	90223299	CLR, iHS	—
5	49828552	50428269	CLR, iHS	*LOC101105495, DIAPH1, HDAC3, RELL2, FCHSD1, ARAP3, PCDH1, KIAA0141, PCDH12, RNF14, GNPDA1*
5	52326560	52893805	CLR, iHS	*YIPF5, KCTD16*
6	28000000	28085452	CLR,iHS	—
6	28085453	28648925	ROH, CLR, iHS	*PDHA2*
6	28648926	29100000	ROH, iHS	*UNC5C*
6	29315643	29699999	ROH, CLR	*BMPR1B*
6	29700000	30805907	ROH, CLR, iHS	*PDLIM5, HPGDS, SMARCAD1, LOC101102814, ATOH1*
6	30805908	31800000	ROH, iHS	*GRID2*
6	31900000	32800000	ROH, iHS	*GRID2*
6	33700000	34295572	ROH, iHS	*CCSER1*
6	34295573	34772184	ROH, CLR, iHS	*MMRN1*
6	34772185	35300000	ROH, iHS	*SNCA*
6	35600000	36022219	ROH, iHS	*FAM13A, HERC3, NAP1L5*
6	42610379	43500000	CLR, iHS	*PPARGC1A*
6	44444781	44542062	CLR, iHS	*SEPSECS*
6	77641559	77987951	ROH, CLR, iHS	—
6	77987952	78100000	ROH, iHS	—
6	78200000	79100000	ROH, iHS	—
6	79200000	80300000	ROH, iHS	*TECRL*
6	103788747	104174840	CLR, iHS	STX18
6	104469916	104600000	CLR, iHS	SLC2A9
7	50265905	50300000	CLR, iHS	*FGF7, ATP8B4, DTWD1, FAM227B, GALK2, COPS2*
7	57347480	58100000	CLR, iHS	—
7	62102120	62296085	CLR, iHS	*DUOX2, SLC28A2, SHF, DUOX1, DUOXA1, DUOXA2, SORD*
7	89789096	90000000	CLR, iHS	—
8	3155608	4000000	CLR, iHS	—
8	32300000	33138639	CLR, iHS	*HACE1*
9	6400000	6485394	CLR, iHS	—
9	18946501	19200000	CLR, iHS	*LOC106991337*
9	37059198	37125877	CLR, iHS	*FAM110B*
10	35582489	35800000	ROH, iHS	*FGF9, MICU2, ZDHHC20, MRPL57, SKA3, SAP18*
10	36600000	38200000	ROH, iHS	*PARP4, CENPJ, RNF17, ATP12A*
10	38597569	38741781	ROH, CLR	—
10	41196220	42600000	ROH, iHS	—
10	43300000	45476505	ROH, iHS	*KLHL1*

10	57631958	58400000	CLR, iHS	—
12	15434575	15561433	CLR, iHS	—
12	62400000	62850420	CLR, iHS	*NMNAT2, SMG7, NCF2, ARPC5, RGL1, APOBEC4*
15	15110855	15349036	CLR, iHS	*AMOTL1, PIWIL4*
18	54152932	54179251	CLR, iHS	*MAP1A, PPIP5K1*
20	8400000	8642666	CLR, iHS	*LOC101115236, NUDT3, RPS10, PACSIN1*
X	86212844	86500000	ROH, iHS	—
X	88600000	88721553	ROH, iHS	*GLUD1*

* Chr, Chromosome.

Table S5. Functional enrichment analysis of candidate genes simultaneously identified by at least two selection signature methods in Hu sheep.

Terms	Term ID	Term Name	*p*-value	Genes
GO_BP	GO:0006590	thyroid hormone generation	0.0034	*DUOX1, CTSK, DUOX2*
GO_BP	GO:0042554	superoxide anion generation	0.0059	*DUOX1, NCF2, DUOX2*
GO_BP	GO:0007215	glutamate receptor signaling pathway	0.0136	*GNAQ, SLC1A1*
GO_BP	GO:2000243	positive regulation of reproductive process	0.0204	*FGF9, UNC5C*
GO_BP	GO:0006909	phagocytosis	0.0214	*CDC42SE1, NCF2, MERTK*
GO_BP	GO:0051603	proteolysis involved in protein catabolic process	0.0251	*CTSK, NRDC, CTSS*
GO_BP	GO:0048488	synaptic vesicle endocytosis	0.0292	*NLGN1, PACSIN1, SNCA*
GO_BP	GO:0006020	inositol metabolic process	0.0337	*PPIP5K1, SLC5A3*
GO_BP	GO:0001508	action potential	0.0404	*GNA14, GNAQ*
GO_BP	GO:0006979	response to oxidative stress	0.0460	*RCAN1, DUOX1, DUOX2*
GO_BP	GO:0086005	ventricular cardiac muscle cell action potential	0.0469	*KCNE1, KCNE2*
GO_BP	GO:0097623	potassium ion export across plasma membrane	0.0469	*KCNE1, KCNE2*
GO_BP	GO:0006559	L-phenylalanine catabolic process	0.0469	*HGD, PAH*
GO_BP	GO:0010463	mesenchymal cell proliferation	0.0469	*FGF7, FGF9*
GO_BP	GO:0060158	phospholipase C-activating dopamine receptor signaling pathway	0.0469	*GNA14, GNAQ*
GO_CC	GO:0016324	apical plasma	0.0002	*DUOX1, KCNE1, KCNE2,*

		membrane		*CTSK, SLC1A1, SLC5A3, DUOX2, PROM2*
GO_CC	GO:0043020	NADPH oxidase complex	0.0023	*DUOX1, NCF2, DUOX2*
GO_CC	GO:0043025	neuronal cell body	0.0034	*MAP1A, SLC1A1, KLHL1, UNC5C, SNCA*
GO_CC	GO:0005737	cytoplasm	0.0294	*RNF14, SKA3, TRHDE, SH3RF1, NUDT3, FGF7, HERC3, FGF9, FAM110B, NPHP1, CDC37L1, TNFAIP8L2, ANXA9, LOC105609508, EPS15, LOC101115261, PACSIN1, SNCA, CDKN2C, PARP4, HGD, KLHL1, ARNT, ARAP3, ANXA10, ARPC5, MERTK, SEPSECS, RCAN1, DIAPH1, ACAP2, GNPDA1, COPS2, GALK2, BNIPL*
GO_CC	GO:0009986	cell surface	0.0356	*DUOX1, NLGN1, KCNE2, SLC1A1, ANXA9, PROM2*
GO_MF	GO:0015459	potassium channel regulator activity	0.0009	*DPP10, KCNE1, KCNE2, SLC5A3*
GO_MF	GO:0005509	calcium ion binding	0.0086	*DUOX1, MMRN1, LOC105609515, LOC101105495, ANXA10, MICU2, PCDH12, ANXA9, PCDH1, EPS15, FSTL1, DUOX2*
GO_MF	GO:0032027	myosin light chain binding	0.0209	*SPATA6L, SPATA6*
GO_MF	GO:0005515	protein binding	0.0284	*LRRC58, PIWIL4, GABPB2, NCF2, LRRK2, TMTC2, SMG7, FSTL1, SH3RF1, LRRTM4, NPHP1, PACSIN1, HACE1, SETDB1, MUC19, KLHL1, ARAP3, UNC5C, MERTK, TTC39A, FCHSD1, ACAP2, MMRN1, COPS2, KCTD16*
GO_MF	GO:0019001	guanyl nucleotide binding	0.0414	*GNA14, GNAQ*
GO_MF	GO:1902282	voltage-gated potassium channel activity involved in ventricular cardiac muscle cell action potential repolarization	0.0481	*KCNE1, KCNE2*
GO_MF	GO:0016174	NAD(P)H oxidase H2O2-forming activity	0.0481	*DUOX1, DUOX2*
KEGG	oas04810	Regulation of actin	0.0093	*FGF7, ITGA4, ARPC5, FGF9,*

		cytoskeleton		*DIAPH1*
KEGG	oas01100	Metabolic pathways	0.0159	*PDHA2, NDUFB4, CERS2, HIBADH, GNPDA1, GLUD1, TXNDC12, SORD, GDA, SEPSECS, PAH, NMNAT2, HGD, ATP12A, HPGDS, BDH1*
KEGG	oas04144	Endocytosis	0.0205	*EPS15, ARAP3, ARPC5, ACAP2, ZFYVE9*
KEGG	oas04922	Glucagon signaling pathway	0.0256	*PDHA2, GNAQ, PPARGC1A*
KEGG	oas04015	Rap1 signaling pathway	0.0367	*FGF7, ARAP3, GNAQ, FGF9*
KEGG	oas00400	Phenylalanine, tyrosine and tryptophan biosynthesis	0.0391	*PAH*
KEGG	oas04730	Long-term depression	0.0459	*GNAQ, GRID2*

* BP, biological process; CC, cell component; MF, molecular function.

Table S6. Descriptive statistics for the litter size of Hu sheep

**Trait**	**Mean**	**SD**	**Minimum**	**Maximum**	**CV(%)**
Litter size	2.36	0.85	1	7	36.02

* SD, standard deviation; CV, coefficient of variation.

Table S7. Significant SNPs associated with litter size in Hu sheep.

**Chr**	**SNP Name**	**Position (bp)**	***p*-value**	**Genes**
14	DU456953_342.1	51891040	2.08E-06	*LOC101112590, CLPTM1, RELB, CLASRP, ZNF296, GEMIN7, PPP1R37, NKPD1, TRAPPC6A, BLOC1S3, EXOC3L2, LOC101115564, MARK4, CKM, KLC3, ERCC2, PPP1R13L, LOC106991561, CD3EAP, ERCC1, FOSB, RTN2, PPM1N, VASP, OPA3*
6	Booroola_FecB(B)	29315643	9.90E-06	*UNC5C, BMPR1B*

* Chr, Chromosome.

Table S8. GO and KEGG enrichment analysis of candidate genes associated with litter size in Hu sheep.

**Terms**	**Term ID**	**Term Name**	***p*-value**	**Genes**
GO_BP	GO:0006357	regulation of transcription by RNA polymerase II	0.0223	*ZNF296, PPP1R13L, ERCC2, FOSB, RELB*
GO_BP	GO:0006289	nucleotide-excision repair	0.0318	*ERCC1, ERCC2*
GO_MF	GO:0003684	damaged DNA binding	0.0324	*ERCC1, ERCC2*
KEGG	oas03420	Nucleotide excision repair	0.0009	*ERCC1, ERCC2*
KEGG	oas04380	Osteoclast differentiation	0.0067	*RELB, FOSB*
KEGG	oas04360	Axon guidance	0.0130	*UNC5C, BMPR1B*
KEGG	oas03022	Basal transcription factors	0.0433	*ERCC2*
KEGG	oas05030	Cocaine addiction	0.0442	*FOSB*

KEGG	oas03460	Fanconi anemia pathway	0.0468	*ERCC1*

* BP, biological process; MF, molecular function.
